# A Qualitative Study: What Do Nurses in Charge in Emergency Departments Do?

**DOI:** 10.7759/cureus.17912

**Published:** 2021-09-12

**Authors:** Shreya Singhal, Ian Hosking, James Ward, Adrian A Boyle

**Affiliations:** 1 Emergency Medicine, Addenbrooke's Hospital, Cambridge University Hospitals NHS Foundation Trust, Cambridge, GBR; 2 Engineering Design Centre, Cambridge University Department of Engineering, Cambridge, GBR

**Keywords:** emergency department, emergency nurse, emergency department operations, nursing leadership and management, nurse in charge

## Abstract

Background

The nurse-in-charge (NIC) role has been implemented in many emergency departments (EDs) to assist with smooth operations and coordination across the ED, together with the emergency physician in charge (EPIC). This work aims to describe the problem-solving approaches used by NICs and the coordination of their role with other team members.

Methods

Observations and semi-structured interviews were performed with NICs in a single centre, where NICs were purposively sampled for a variety of experience levels. During the observations, field notes were taken for every action conducted by the NIC in ED; the semi-structured interviews involved a combination of question prompts and a blank diagram of the ED that the NICs were asked to annotate. Constant comparative analysis based on grounded theory methodology was used for this qualitative study.

Results

Eight different problem-solving approaches were identified. These are placing, targeting, guiding, juggling, chasing, team-leading, escalating and de-escalating. The last three were exclusive to NICs, whereas the others were shared to some degree with the EPIC. Seven team situational awareness processes used by NICs for coordination with other team members were identified, leading to a discussion on team synchronisation and shared awareness mechanisms. In particular, shared internal models amongst the NICs and also other team members provide a framework for analysing how team members function together in a healthcare setting.

Conclusions

Emergency department NICs have a number of problem-solving approaches that have been defined and shown to have a degree of overlap with the emergency physician in charge. Shared awareness between the NIC and other ED team members facilitate decision-making and smooth coordination. These findings provide a better understanding of the role of the NIC and are useful for describing solutions for patient flow.

## Introduction

The nurse in charge (NIC) in the emergency department (ED) is a role implemented to provide a variety of functions aiming for smooth operations and coordination across the department, in close coordination with the emergency physician in charge (EPIC). The nurse in charge is expected to be responsible for all nursing care in the emergency department [1]. This is a demanding role and there is little published literature about how the role is delivered.

The aim of this study was to describe the problem-solving approaches, i.e. heuristics [2], used by NICs in order to meet the aims of their role, as well as to provide a better understanding of the same. This information would be useful for training nurses, as well as providing a baseline for future work to evaluate best practices. This study is linked to previously published work describing the heuristics of the emergency physician in charge [3].

## Materials and methods

Design

This was a qualitative explorative study using the grounded theory approach [4,5]. Grounded theory is a methodology that aims to discover theories from systematically gathered data, where ideas and concepts are said to emerge from the data through structured analysis. Grounded theory was considered suitable to use for this study for its methodology in exploring little-researched areas to act as a landscape for understanding individuals’ views and interactions; Charmazian theory was used in particular for its subtlety of reflecting the story of participants, which was felt to be particularly relevant in this study involving analysis of the role of the NIC which is carried out in a high-pressure inter-personal environment [5]. Grounded methodology has been used effectively in other studies in a healthcare setting [6-8].

Sample and setting

We performed the study in ED in Addenbrooke’s Hospital, Cambridge, England in February 2020. The distribution of experience/pay across the nursing cohort in the ED was 12.92 WTE Band 7, 31.07 WTE Band 6 and 84.52 WTE Band 5 (1 WTE = 1 Whole Time Equivalent = 37.5 hours); all NICs were Band 7 nurses. Participants were purposively sampled [9] for a variety of experience levels amongst the team of NICs and, as the study progressed, sampling was done until thematic saturation was achieved [10]. Prior to commencement, the NIC team were made aware of the study by email, including the reasons for the research and to introduce the author SS as the lead field researcher. Participants were approached and consented face-to-face at the time of the study.

Consent was gained from all participant NICs. Ethical approval was not required, as described by the NHS Health Research Authority Approval Flowsheet.

Data collection

The observation period was defined following a pilot study: an equal split of 'in' and 'out of hours' was selected, where 'in hours' was defined as 8 am-8 pm weekday shifts, and 'out of hours' was defined as 8 pm-8 am and weekend shifts. Observation periods were a minimum of two hours, which was felt to be the minimum required to gather sufficient data during the pilot study, and start times were spread evenly throughout the shift in order to maximise the range of hours observed. During the observation period, SS made detailed field notes of every action conducted by the NIC in ED that was observed, where an action was defined as any task, movement or interaction considered relevant to the study. SS also asked questions of the NICs during the observation period to better understand their intentions and thought processes.

Secondly, semi-structured interviews were conducted, where interviewees were prompted with questions. All interviews were started with the question ‘What is the aim of the role of the Nurse in Charge?’, and then participants were asked to elaborate on specific issues as they arose, in line with the constant comparison technique [4,11]. Some examples of other questions asked during the interviews include ‘Who do you interact with in your role?’, ‘What makes a good NIC?’ and ‘When are you most effective at your job?’; the follow-up questions during the interviews were not prescribed in advance, but rather followed the natural course of a discussion, in line with grounded theory. The NICs taking part in the semi-structured interviews were asked to complete a template diagram (a blank version of Figure 2), designed by IH. They were also shown the table of EPIC heuristics developed by Hosking et al. [3] and asked to highlight which they felt they also did, along with examples; this was done at the end of the interview so that the EPIC heuristics shown to them did not bias their thoughts or responses for the rest of the interview. The pilot carried out was considered to be suitable and so was included for analysis. Audio recordings of the interviews were taken with participants’ consent, and field notes were taken by SS. Where notes had gaps, this was noted during the time of interview and the recordings were listened to afterwards to fill in the gaps in notes.

Analysis

Analysis was conducted according to the constant comparative method [4,11] as part of the grounded theory methodology [4,5]. This involved initial coding by SS, which was constructed by analysing all notes from observations and interviews, and began from the first interview. SS annotated the notes in the margins after each observation/interview, writing a descriptive phrase for each action in the notes. Focused coding enabled sorting and grouping of similar descriptive phrases into several core categories from the initial codes, and was iterated for each subsequent participant. Later data collected from new interviews were used to refine the constructed categories and participants in later interviews were shown initial results for their feedback, until thematic saturation was reached [5]. Building on the research by Hosking et al. [3], the themes identified provided a basis for the final identification of themes in this study. Memo-writing was employed throughout to help analyse and conceptualise the data.

Addressing bias

A certain amount of inherent bias is acknowledged with the described methodologies. The following steps were taken in an attempt to minimise bias: AB (employed as ED-EPIC) was not involved in data collection and coding in order to remove any bias from working in close proximity with the participants; data analysis was discussed and agreed with the research team at multiple stages of its development; themes from Hosking et al. [3] were used for final categorisation, but not in the coding stages so that all relevant data was included for analysis.

## Results

Eight NICs with a broad range of experience levels were approached and all gave consent to be participants. Observations were carried out across 21 hours in total, with an equal split of in and out of hours. Furthermore, the semi-structured interviews lasting 30-45 minutes were conducted with four NICs from the eight participants; none refused to take part and there were no repeat interviews. Four out of eight NICs were interviewed due to availability.

As described in the methods section, focused coding was used to generate core categories, which are called the problem-solving approaches or ‘heuristics’ of NICs, as shown in Table 1. This work is related to the initial studies done by Hosking et al. [3], in which nine heuristics of an emergency physician in charge (EPIC) were developed by qualitative studies. The examples in Table 1 were identified during the observation period through initial coding. To illustrate the coding methodology, the following gives an illustrative example of the process from written notes to heuristic: 'NIC 1 discussing with charge nurse of resus area which potential patients can be stepped down in case a more critical patient is brought in by ambulance' (written note); 'Stepping down patients from resus' (initial code); 'Moving patients to a different area' (focused code); 'Placing' (heuristic). In this manner, all written notes were grouped into initial codes provided as examples in Table 1, and thus subsequent heuristics were developed.

**Table 1:** Problem-solving approaches used by nurses in charge in ED * The hospital site matron has the role of coordinating between EPIC/NIC, the operations centre and bed manager. They have a focus on managing bed capacity and patient flow, in relation to the hospital's key performance and quality standards. EPIC: emergency physician in charge; NIC: nurse in charge

Heuristic	Definition	Examples
Placing	Moving patients to a different area, either to improve the appropriateness of care or to free up specific resources	Focus on high acuity patients (‘who are you worried about?’); constant view of ambulance offloading and available space for them; stepping down patients from resus
Targeting	Putting specific resources into an area to help flow	Put most skilled nursing staff in charge of resus and ambulance bay first
Guiding	Advising staff	Prompting charge nurse of area to reallocate front door staff to booking in rather than doing secondary observations when pts queueing at the front door; long term mentoring; staff morale – conflicts between staff/patients, enthuse/encourage
Juggling	Moving resource around to alleviate bottlenecks	Moving staff from a quiet area to the front door when a queue is building up; closing one area in times of low staff (2-6 am)
Chasing	Chasing investigations and consultations and decisions from inpatient teams. Managing dissent	Chasing porters for patient transport, by radio call or in-person conversation; chasing specialist consultation; asking bed manager to prioritise patients who are ready to move to the ward rather than those with bed requests
Team leading	Judging and coordinating staff rota – requires a good knowledge of skill mix and individual staff	Always have in mind who can cover in case staff need to be reshuffled mid-shift; managing staff off sick and on breaks
Escalating	Escalation to hospital site matron* and operations centre	Asking hospital site matron if they are happy to open PAT1/2 (ambulance bay)
De-escalating	Difficult incident management	ED-skilled to manage major traumas, arrests and difficult mental health patients, particularly when more junior nurses are struggling/need more help

**Figure 1:**
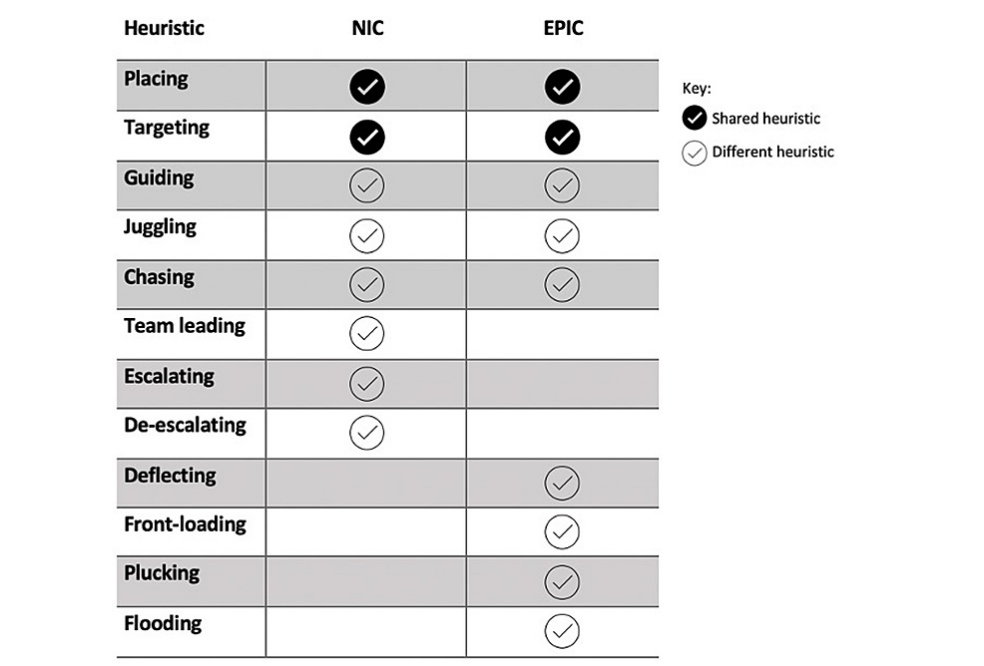
A comparison of EPIC and NIC heuristics Constructed using information from Table 1, and the first table from Hosking et al., 'What do emergency physicians in charge do?' [3]. The heuristics identified as different between EPIC and NIC are based on how they carry out the heuristics. For the full table, please refer to the Appendix. EPIC: emergency physician in charge; NIC: nurse in charge

**Figure 2:**
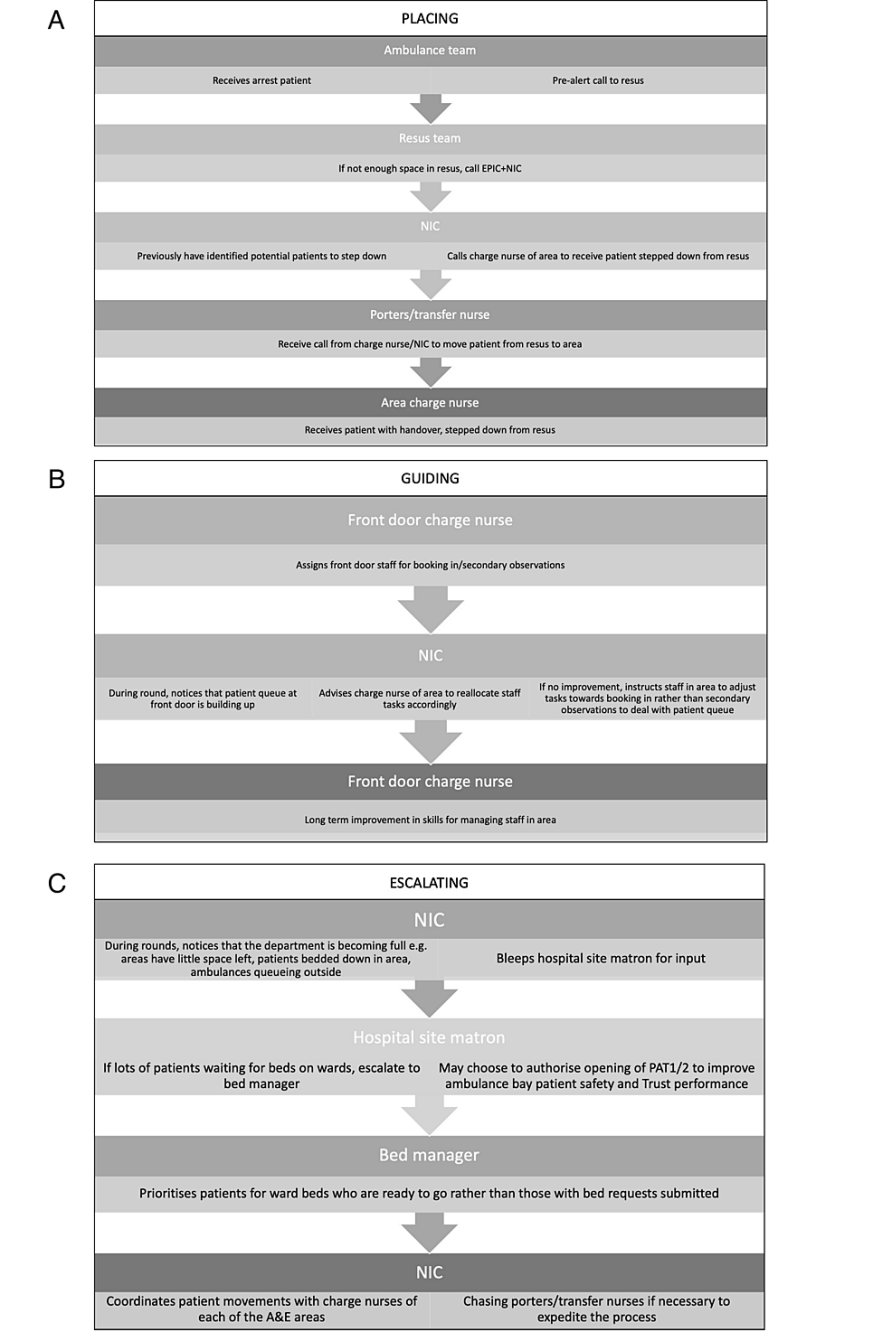
Flow diagrams demonstrating examples of heuristics defined in Table 1. The sub-figures demonstrate the following heuristics: a) placing, b) guiding, c) escalating. EPIC: emergency physician in charge; NIC: nurse in charge

Figure 1 compares the heuristics used by NICs versus EPICs (a detailed comparison is provided in the Appendix). Figure 2 provides flow diagrams showing examples of how the heuristics were carried out in practice, as well as the relevant team members that the NIC interacted with in order to complete the task. A full map of stakeholders relevant to the NIC role according to the observational studies is provided in the Appendix.

**Figure 3:**
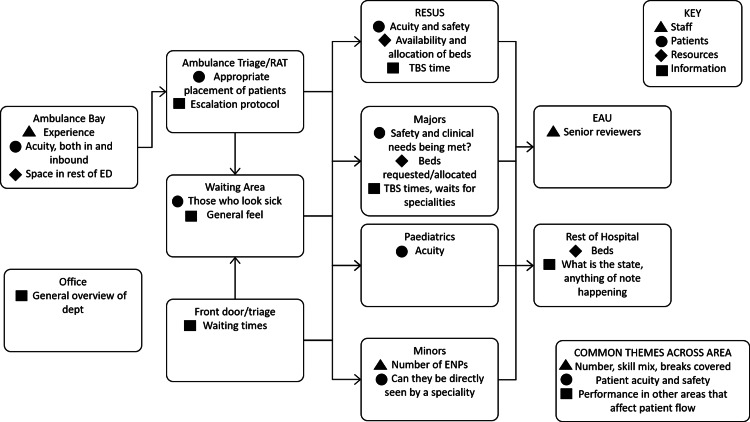
Schematic indicating what the NIC looks for in each area of ED when doing their 'rounds' TBS time: to be seen time; ENP: emergency nurse practitioner

In addition to the heuristics used by NICs, Figure 3 provides a visual schematic of what the NIC looks for in each area during their ‘rounds’ of ED, where ‘rounds’ describes the act of walking around the various areas of the ED in a systematic order, as well as looking at the Electronic Health Record and Trackboard (patient information and tracking system for the ED). Each NIC had a different style of carrying out their rounds - varying in terms of speed, content, order of visiting the areas, how formal/informally it was done and whether they did it with/without the EPIC and hospital site matron. Despite variations in their styles, the NICs arrived at the same conclusions about the key knowledge areas for each area that was necessary for them to effectively carry out their role. The information in Figure 3 summarises findings from both the observational studies and the semi-structured interviews (see Appendix for examples of template diagrams completed by NICs during interviews).

**Figure 4:**
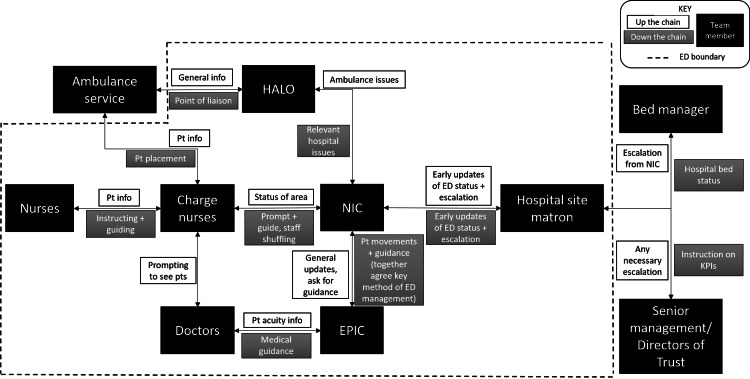
Flow of information between key team members relevant to the NIC role From left to right is an approximate indication of increasing seniority HALO: Hospital Ambulance Liaison Officer; Pt = patient; KPIs = key performance indices; EPIC: emergency physician in charge; NIC: nurse in charge

As previously stated, the role of the NIC does not stand in isolation, but instead in close connection with other team members. In order to describe these relationships, Figure 4 is the result of examination of the interactions between team members and NICs during the observational studies, and the key purpose of communication between each of the members.

The model of team situational awareness as described by Endsley and Jones has been used as a basis for elaborating the dynamics between team members in the ED in this study [12]. The information in Table 2 summarises key themes that emerged from the interview and observational studies in relation to team situational awareness.

**Table 2:** Team situational awareness between NIC and other team members Adapted from Endsley and Jones' model of team situational awareness [12]. Self-checking = checking with relevant team members that they shared the same picture of the situation; failure to prioritise = team members following their own direction and priorities, or losing track of the main goal. EPIC: emergency physician in charge; NIC: nurse in charge

Team situational awareness mechanisms and processes	Notes and examples
Coordination: Verbal in-person, bleeps and telephone when not in same physical space	Instigated by NIC to EPIC E.g. Change in management of ambulance bay; information about patient movements
Visual displays: Electronic Health Record and Trackboard	EPIC more interested in details of patients in resus; NIC looked more heavily at wait times in all areas of ED
Shared environment: EPIC found in resus NIC in all areas	EPIC in resus with the purpose that the sickest patients are there and so need most immediate attention
Self-checking: Up the chain of hierarchy (see Figure 4)	E.g. NIC decided which patients they would like to move, and would check this with EPIC
Coordination: Rounds at the start of the shift with hospital site matron, and sometimes joined by EPIC. Periodic coordination between NIC/EPIC/hospital site matron during shift	Whether EPIC joined the depended on their mutual working style as a NIC/EPIC team
Prioritisation: Contingency plans already in place	E.g. when the department became full and patients needed to be diverted to other hospitals, the process was known in advance [13]
Failure to prioritise: Conflict of priorities between different stakeholders	E.g. Directors and NIC/EPIC conflicted with prioritising patient safety versus hospital targets/requirements; EPIC wanting the corridor open versus NIC not because of staffing issues

## Discussion

Eight heuristics of the nurse in charge role in the emergency department have been described, as well as the key aspects during NIC rounds, the flow of information between the NIC and team members, and a view on their team situational awareness.

The NIC problem-solving approaches

Elements of the eight heuristics proposed in Table 1 have been reported in previous literature studying the charge nurse role. Connelly et al. [14] described competencies in the domains of clinical, critical thinking, organisational and human resources; Jasper et al. [15] identified three common themes - managing unit performance, managing people and resources, and empowerment of self and others; Sherman et al. [16] discussed five common leadership qualities of charge nurses in acute care environments - managing communication, acting as a team coach, being seen as approachable, working like an air traffic controller and being viewed as a professional. The similarities seen in research on charge nurses compared with the reported heuristics in this study give weight to our findings. Despite the nurse in charge role in ED being relatively new, commonalities with pre-existing charge nurse roles give confidence that those assuming the NIC role should have developed similar qualities and problem-solving approaches having worked previously as charge nurses. This is crucial for decision-makers to trust the ability of the NIC to improve and foster patient flow through the ED and act as the close liaison to EPIC that it is designed to be [1].

Furthermore, a comparison between NIC and EPIC heuristics provides some noteworthy outcomes for discussion (see Figure 1 and table in the Appendix). The role of the EPIC has been described by the Royal College of Emergency Medicine as 'Command and Control' [17]. Hosking et al. [3] described nine heuristics used by the EPIC, which goes into further detail about their role; little other research clearly gives findings regarding the EPIC role. Comparing the NIC and EPIC heuristics, 'placing' and 'targeting can be seen to be reasonably similar between NICs and EPICs, each enacting via their own staff team (nurses and doctors, respectively) for the purposes of patient flow and safety. However, the remaining heuristics differ for NIC and EPIC in that the NICs have a focus on operational opportunities for patient flow, and the EPIC focuses on the clinical aspects of patient flow. This operational versus clinical focus is an important nuance between the NIC and EPIC, providing insight into their complementary roles. 

Situational awareness models

The idea of the situational awareness model has been described by Endsley and Jones as the perception of the elements in the environment within a volume of time and space, the comprehension of their meaning, and the projection of their status in the near future' [12]. Similarly, shared mental models describe the overlap or co-understanding between individuals, and have been used to describe the ability of team members to think in a similar way [18]. This idea of a shared understanding of the situation has been described in healthcare research [19-22], and it is a valuable framework for analysing how team members function together in healthcare settings.

The NICs as a Team

Concept mapping has been used in the literature as a method of capturing team mental models [23,24]. We found that the NICs have a shared mental model, as depicted in Figure 3. The fact that they all look for the same information in each area of ED in order to carry out their job adds strength to our answers to the question of this study: 'What do the Nurses in Charge in ED Do?'.

The Operational Team in ED

In the ED, the NIC very rarely functions as an individual, but rather in close proximity to other key stakeholders in the system (Figure 4). The mental model, therefore, is shared not only between NICs, but also certain aspects are shared between team members (see table in Appendix), facilitating effective team-working which is vital for patient safety and outcomes [25]. Again, the complementarity between NIC and EPIC becomes evident - for each shared mechanism, each has their own role or specific mannerism within it. There was a status quo established, creating a sense of predictability. In the Emergency Department, where it is anybody's guess as to who or what will enter the front door/by ambulance, this predictability between team members provides stability amongst the chaos. It allows team members to rely on each other and streamlines efforts without having to spend valuable time discussing the ways in which they will work together, known as cognitive unloading [26]. Self-checking, coordination and prioritisation are processes used by effective teams [12], supporting the positive impact of the NIC role as part of the ED operational team. Conflicts between mental models ('failure to prioritise', Table 2) were rare in the period of observation and were typically addressed informally. The resolution of conflicts was based on hierarchy when faced between hospital directors and NIC/EPIC; although EPIC and NIC are designed as equal roles, conflicts between EPIC and NIC concluded with the EPIC getting the result they wanted, indicating an implicit hierarchy.

Limitations of the study

There are some important limitations to this work. This study was conducted in a single centre and it is uncertain whether our results are valid elsewhere. The sample size was limited (noting that there were a total of 12.92 WTE Band 7 nurses employed by the ED, out of which eight took part in the observational study and four in the interviews) and data collection was constrained by the COVID-19 pandemic.

However, previous work of this nature has employed similar methodologies and the results have high face validity (Hosking et al. [3]). Furthermore, whilst acknowledging the limited number of interviews conducted, their responses correlated well with the observations, adding confidence in the fact that the results were formed through two different mechanisms of data collection. The importance of this work is that it provides an initial understanding and analysis of the role of the NIC, which can then be used as a basis for studies of larger sample sizes performed at multiple centres. 

## Conclusions

The components of the nurse in charge role have been described and distilled into eight heuristics, with the main aim of facilitating patient care by improving operations and patient flow within the ED. In addition to these problem-solving approaches, it was discovered that there is not only a shared mental model of information within the ED between different NICs, but also between NICs and EPICs, which is essential for the smooth running of the ED. The shared awareness amongst the team is facilitated both formally and informally; formally, by the implementation of shared IT systems and a shared physical environment, and informally by the mutual understanding of the status quo.

## Figures and Tables

**Figure 5:**
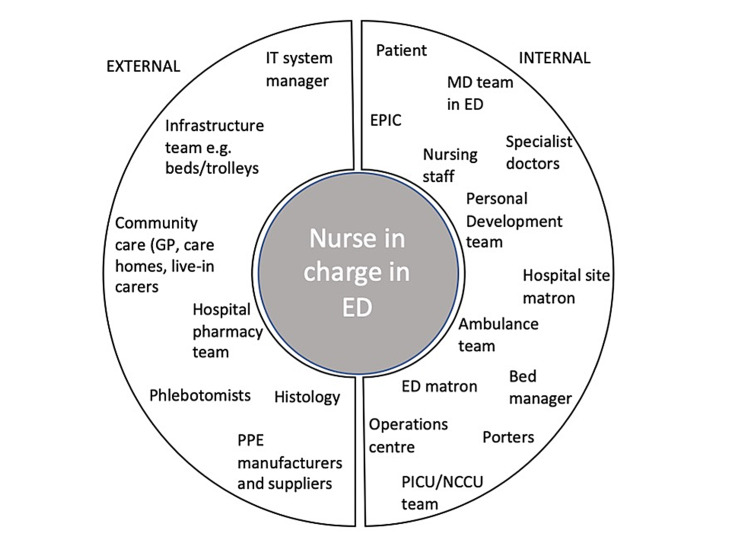
Map of stakeholders related to the NIC in ED Internal stakeholders are defined as those with whom the NIC directly interacted, and external stakeholders are those that the NIC didn’t directly interact with, but were mentioned/considered in conversations. All team members included in this map were seen or mentioned during the observational studies.

**Figure 6:**
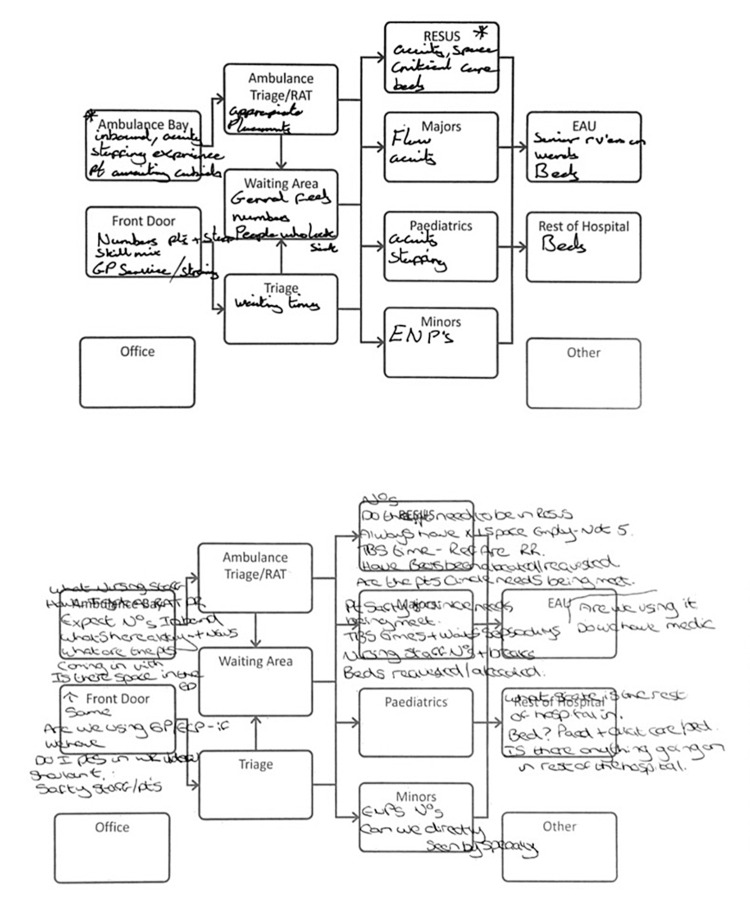
Examples of template diagrams completed by NICs during the semi-structured interviews

## Appendices

Table 3 contains a detailed comparison of the heuristics used by NICs versus EPICs, previously referenced in Table 1:

**Table 3:** A comparison of EPIC and NIC heuristics Constructed using information from Table 1 and the first table in Hosking et al., 'What do emergency physicians in charge do' [3]. Text in italics indicates the heuristics that are considered to be the most similar for NICs and EPICs.

Heuristic	Definition	NIC Examples	EPIC Examples
Placing	Moving patients to a different area, either to improve the appropriateness of care or to free up specific resources	Focus on high acuity patients (‘who are you worried about’); constant view of ambulance offloading and available space for them; stepping down patients from resus	Identifying which patients who have arrived by ambulance can sit in the waiting room, or identifying which patients can go to the observation ward
Targeting	Putting specific resources into an area to help flow	Putting most skilled nursing staff in charge of resus and ambulance bay first	Placing a senior doctor into an area of low acuity to efficiently see lots of patients
Guiding	Advising staff	Prompting charge nurse of area to reallocate front door staff to booking in rather than doing secondary observations when pts queueing at the front door; long-term mentoring; staff morale – conflicts b/w staff/patients, perk up in general	Advising junior clinical staff which patients can be sent home safely and which need to be admitted
Juggling	Moving resources around to alleviate bottlenecks	Moving staff from a quiet area to the front door when a queue is building up; closing one area in times of low staff (2-6 am)	Reallocating a single staff to a resuscitation room case and arranging for another staff member to take on their own work
Chasing	Chasing investigations and consultations and decisions from inpatient teams; managing dissent	Chasing porters; chasing specialist consultation; asking the bed manager to prioritise patients who are ready to move to the ward rather than those with bed requests	Clarifying which inpatient team will take over further care
Team-leading	Judging and coordinating staff rota – requires a good knowledge of skill mix and individual staff	Always have in mind who can cover in case staff need to be reshuffled mid-shift; managing staff off sick and breaks	
Escalating	Escalation to hospital site matron and operations centre	Asking hospital site matron if they are happy to open PAT1/2 (ambulance bay)	
De-escalating	Difficult incident management	ED-skilled to manage major traumas, arrests and difficult mental health patients, particularly when more junior nurses are struggling/need more help	
Deflecting	Triaging a patient to alternative care		Sending a self-presenting patient to an urgent care centre or general practitioner
Front-loading	Organising investigations for patients early on in their ED stay		Ensuring X-rays are organised early for patients with suspected fractures or CTs for patients with head injuries or suspected renal colic
Plucking	Picking out patients that need specific intervention to speed up their progress		Early referral to liaison mental health services for appropriate patients
Flooding	Putting a large number of staff members in an area to empty an area in advance of a surge		Allocating extra staff to the paediatric area to cope with an expected surge of children after school hours

Figure 5 provides a full map of stakeholders relevant to the NIC role according to the observational studies:

Figure 6 contains examples of template diagrams completed by NICs during interviews:
